# Notes from Cornwall

**Published:** 1991-12

**Authors:** David Levine


### From our Correspondents

### NOTES FROM CORNWALL

A well-known maxim, used to put hazards into perspective, reminds the patient that treatment can never be completely safe; there's risk involved even in crossing the road. Whoever invented this saying probably lived or worked in the London borough of Camden.

The trek to London for the autumn meeting of the British Society of Gastroenterology reinforced my view that I'm better out of the big city.

Three nights in a central London hall of residence brought inklings of the loneliness and tedium which must afflict students who spend months at a time in similarly meagre, often undergound rooms. The usual, presumably African, tenant of my cell had left a few clues to his suffering. As a Gastroenterologist I performed the ritual lockerotomy of the under-sink cupboard. Two empty gin bottles and one of "Uganda Wariga" nestled close to two similarly drained containers of Kolanticon gel and one of Gaviscon. So now my educational trip had become a domiciliary visit, albeit in the patient's absence. What to do? Should I leave a selection of $H^2$ blockers from the pharmaceutical stands or just a note with the telephone numbers of some local endoscopy units? Perhaps instead he could seek solace in the imaginative and "discreet" services offered on the printed cards lining the nearby BT telephone box. Those girls could teach the NHS a thing or two about purchasers and providers. The service contracts are clear, the waiting lists short, the customers choose their own treatments and no one gives a toss about extra contractual referrals.

And what has the return to Cornwall had to offer? Hospital staff have, with commendable control, restrained their joy on hearing that our acute unit is to become a Trust.

Every organisation has a limited capacity to accept change. The challenge lies in assessing this capability and knowing what the limitations are. Increasingly though we are asked to take on new schemes and projects, most of them untried, all of them pushed at breakneck speed. Sometimes however, the pace slows dramatically. Nine months ago we were asked to urgently produce "job plans". To date there has been no response to those of us who did as we were asked. We know exactly why and indeed feel some sympathy for local management — but one has to expect problems when a highly publicised exercise is based on a myth.

Clinical directorates are now, it seems, the answer to our problems, but assessing the likely benefits and drawbacks of directorates, and indeed of Trusts, is rather like deciding whether to start using a new drug purely on the basis of the manufacturer's advertisments. What are the advantages on offer? Some say power and control, but even supposing that the pursuit of power is a desirable activity for medical practitioners it is doubtful whether it has any meaning without control of the flow both of money and of work.

Quite properly the public have come to expect an end to health care rationing carried out by doctors "behind closed doors". This begs the question, who will do it and be seen to be doing it? Not apparently the purchasers who should have welcomed the responsibility that goes with the power. Few of the "glossies" tell the public what can't and won't be done. Perhaps it's not too cynical to suggest that the amazingly generous gift of budgets to directorates is a neat way of ensuring that doctors still do the rationing but with the doors open in order that blame is clearly visible.

Whilst Consultants will keep the freedom to be scapegoats for the high cost of patient care, we may not for much longer be allowed to debate the issues openly or advise about that care. Not, that is, if Trust personnel policy and practice follows the formal guidance paper produced, like so many others, by the Trent RHA Document;[1] "Suppose people in key positions manifest a lack of commitment to organisational goals, ideals and values? What then? What about renegades, subversives and opposers of what is being attempted?" *sic.*

Perhaps you have heard this sort of language before. If so, don't imagine for a moment that it is just a clumsy accident. These emotive descriptions of anyone with alternative views are carefully crafted to label such people as requiring scorn or punishment without question. It has always been the language of authority which attempts to justify unacceptable behaviour. It is part of the same corrupt language which talks about "corporate mission statements". Trusts don't have a monopoly on values and ideals but once you have told the Public that your mission is "improved patient care", anything is justified and anyone who questions your aims must by definition be mad or bad.

The abolition of cogwheel may well allow easier executive decision-making. Whether this will be of overall benefit remains to be seen. What will be lost is the chance locally for Consultants in different specialities to understand each others problems, debate the issues and suggest compromises which should provide the best arrangements for clinicians to manage patients.

Unfortunately it's now beginning to sound like a modern fairy story. Perhaps the animals will still assemble on Sunday mornings to salute the flag, sing 'Beasts of England' and receive their orders for the week but there will be no more debates.

### REFERENCE

1. Paper for General Managers of Self Governing Hospital Trusts. Personnel Policy and Practice, The Challenge for S.G.T.s, para 3, 12.

David Levine

